# Supplementary material for: Optogenetic dissection of Rac1 and Cdc42 gradient shaping
Source: Nat Commun. 2018 Nov 16;9:4816. doi: 10.1038/s41467-018-07286-8 (PMC6240110; doi:10.1038/s41467-018-07286-8)
Supplement: Supplementary file 2 — Supplementary Information [file 41467_2018_7286_MOESM2_ESM.pdf]

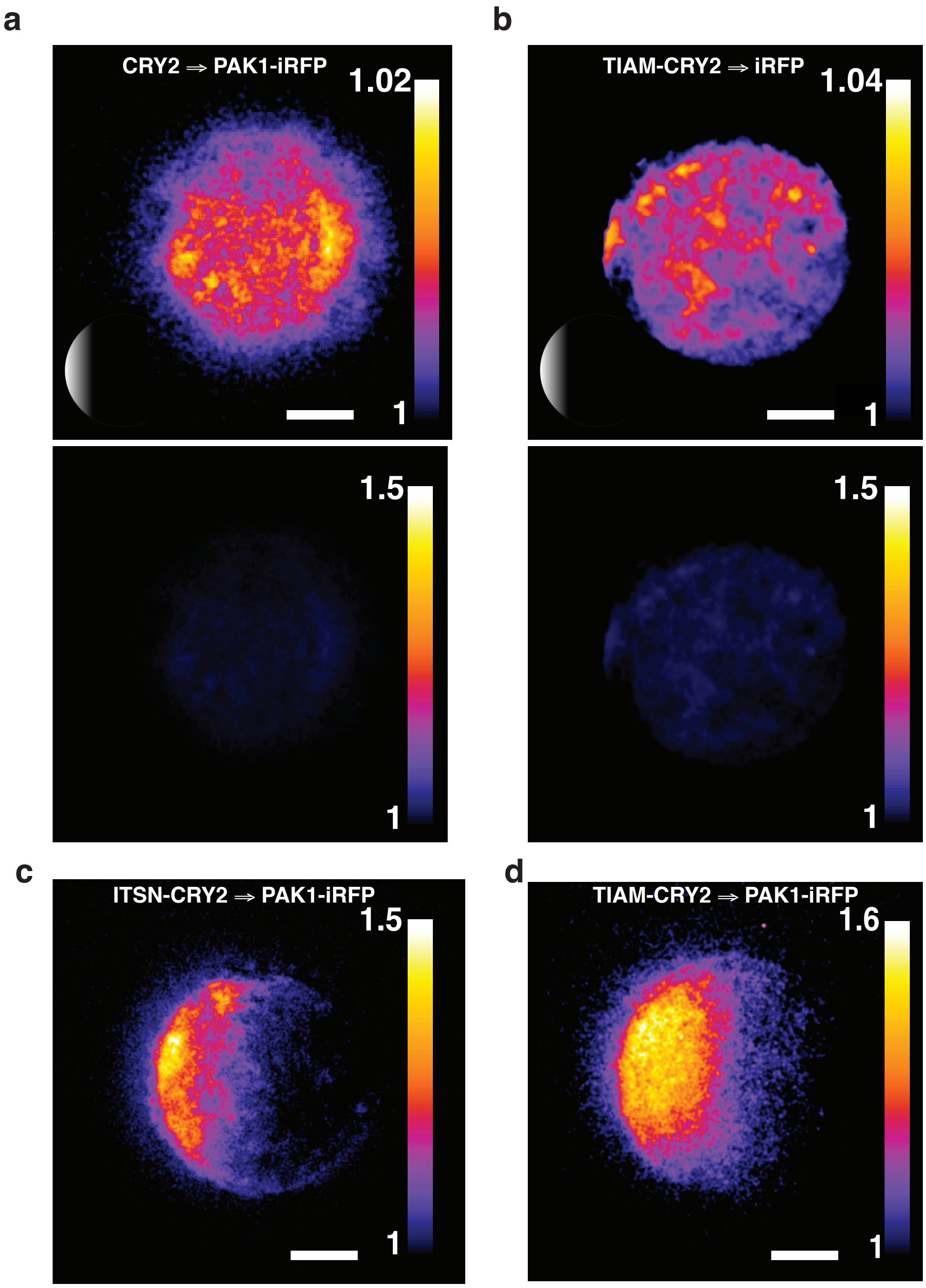

Supplementary Figure 1: Controls for the membrane recruitment of PAK1-iRFP following ITSN-CRY2 or TIAM-CRY2 activation

(a) Membrane recruitment of PAK1-iRFP following 4x activation gradients of CRY2-mCherry. Fluorescence was recorded using TIRFM in HeLa cells on round micro-patterns. Micrographs represent the averaged fluorescence of n=13 cells. **(b)** Membrane recruitment of iRFP following 4x activation gradients of TIAM-CRY2-mCherry. n=13 cells. The color maps represents the fold change of fluorescence, spanning over their actual range (top) or over the same range as in the case of PAK1-iRFP activation by ITSN activation (bottom) for comparison with the effect of ITSN and TIAM (**(c)** and **(d)** respectively, taken from figure 2d and 2f). Insets show the illumination pattern (not to scale). Scale bars: 10 µm.

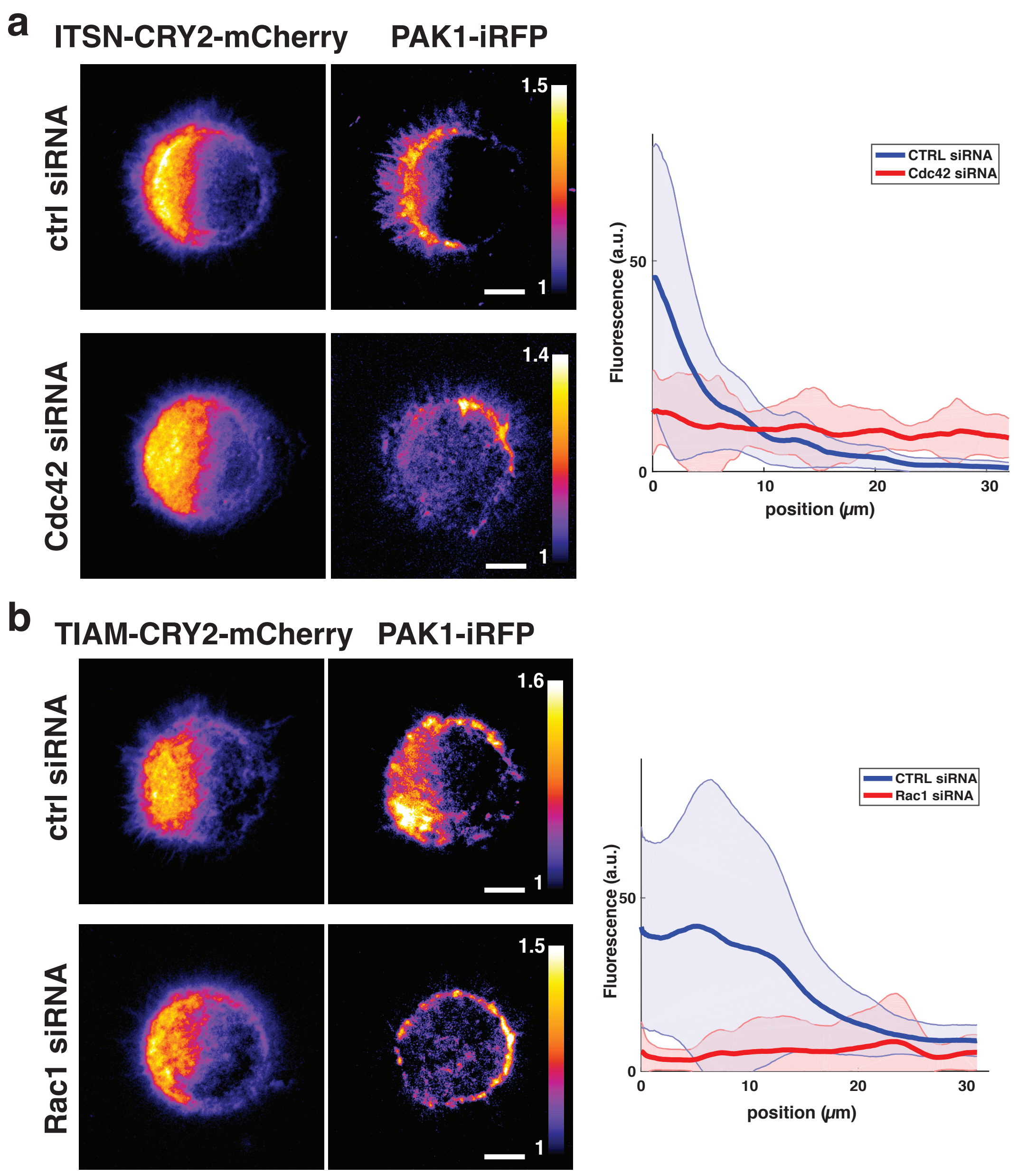

Supplementary Figure 2: specificity of the optogenetics-controlled GEFs ITSN and TIAM
(a) Recruitment of PAK1-iRFP following 4x activation gradients of ITSN-CRY2-mCherry after transfection with control (top, n=12) or Cdc42-specific siRNA (n=11). The curves represent the averaged fluorescence of PAK1-iRFP along cell diameters in both conditions. Error bars: s.d. **(b)** Average recruitment of PAK1-iRFP following 4x activation gradients of TIAM-CRY2-mCherry after transfection with control (top, n=11) or Rac1-specific siRNA (n=9). Error bars: s.d. Scale bars: 10 μm.

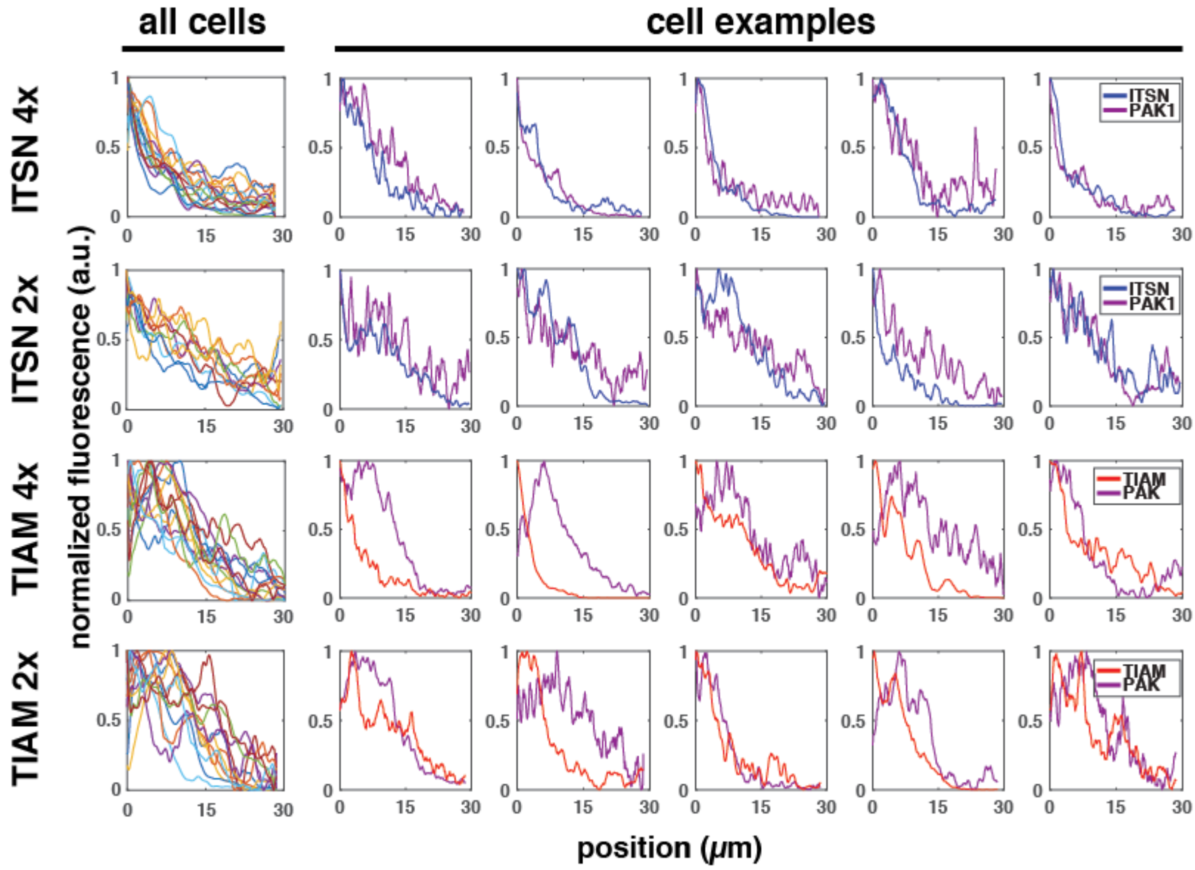

Supplementary Figure 3: Individual curves of PAK1-iRFP recruitment following activation gradients of ITSN and TIAM in individual cells.

PAK1-iRFP fluorescence profiles along cell diameters following 4x or 2x activation gradients of ITSN and TIAM corresponding to the averaged curves presented in Figure 2e and 2g. For each condition, all PAK1-iRFP curves are represented (left column). A few examples from single cells are also depicted (right columns) with the fluorescence ofPAK1-iRFP (purple) compared to ITSN-CRY2-mCherry (blue) or TIAM-CRY2-mCherry (red) in each cell.

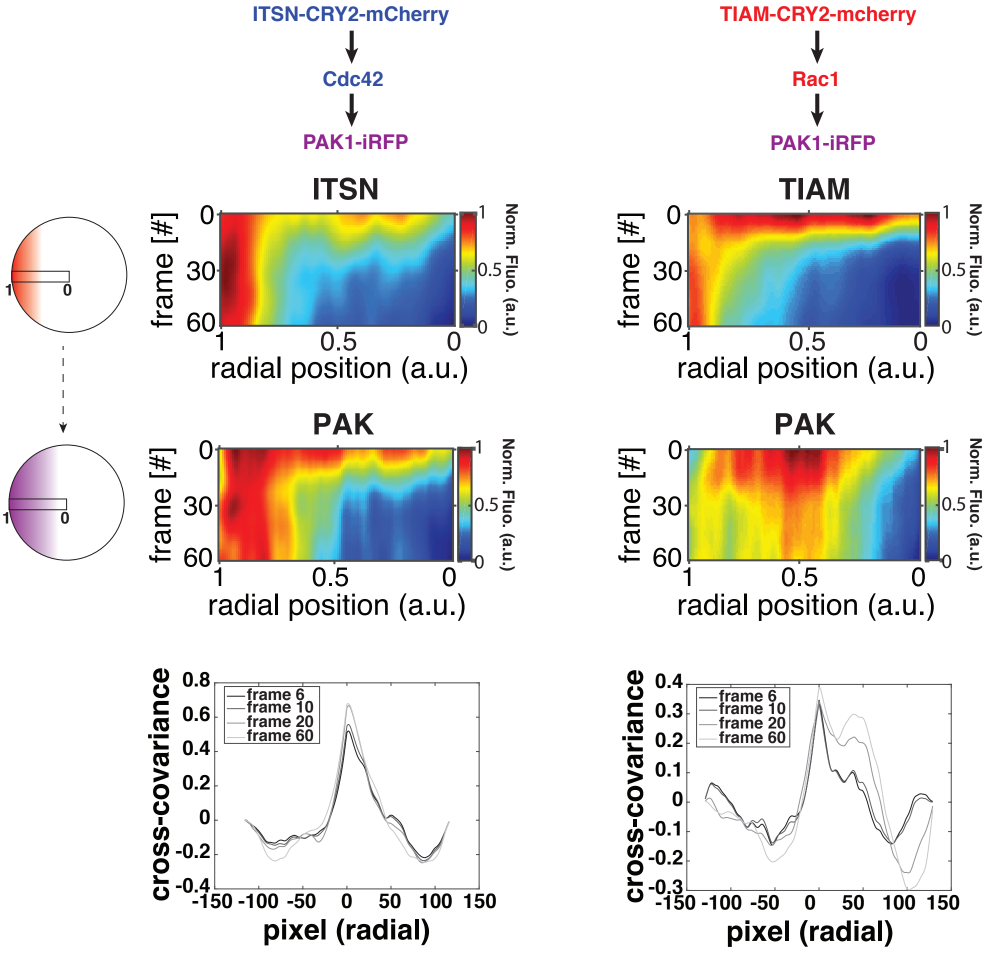

Supplementary Figure 4: temporal analysis of optogenetics-induced Cdc42 and Rac1 gradients

Hela cells expressing CIBN-GFP-CAAX and PAK1-iRFP together with ITSN-CRY2-mCherry (left) or TIAM- CRY2-mCherry (right) adhered on round micropatterns were illuminated with 4x gradients. We quantified the membrane recruitment of CRY2-mCherry fused GEFs (top) and PAK1-iRFP (middle) along cell radii over time. We calculated the cross-covariance between fluorescence signals from each GEF respectively and PAK1 (bottom). n=15 in both cases. Steady-state was reached quickly and the bump shape of Rac1 activity gradients is not a transient state.

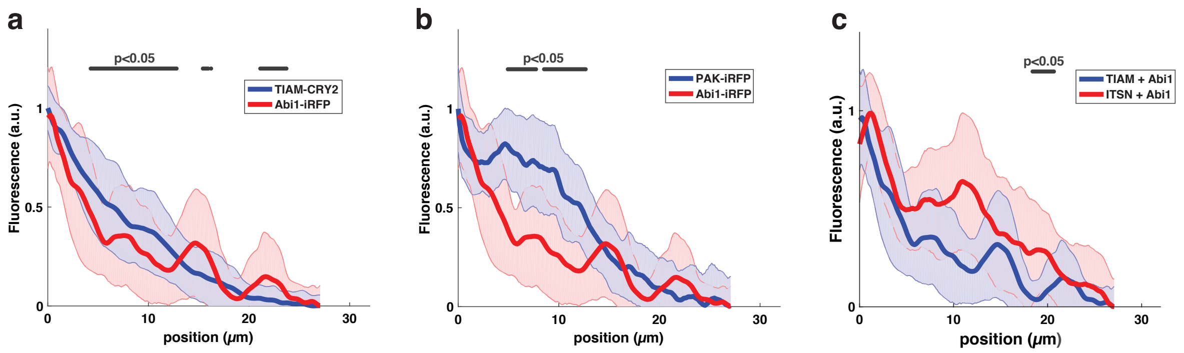

Supplementary Figure 5: Abi1 patterns downstream activation gradients of TIAM-CRY2
(a) Average recruitment of Abi1-iRFP following 4x optogenetic gradients of TIAM-CRY2-mCherry (n=10). **(b)** Recruitment of Abi1-iRFP compared to recruitment of PAK1-iRFP (n=15) measured in similar conditions (stimulation of 4x gradients of TIAM-CRY2-mCherry). **c)** Recruitment of Abi1-iRFP following 4x optogenetic gradients of TIAM-CRY2-mCherry (n=10, blue) or ITSN-CRY2-mCherry (n=11, red). Error bars: s.d. Gray bars: $p < 0.05$ (Wilcoxon rank sum test).

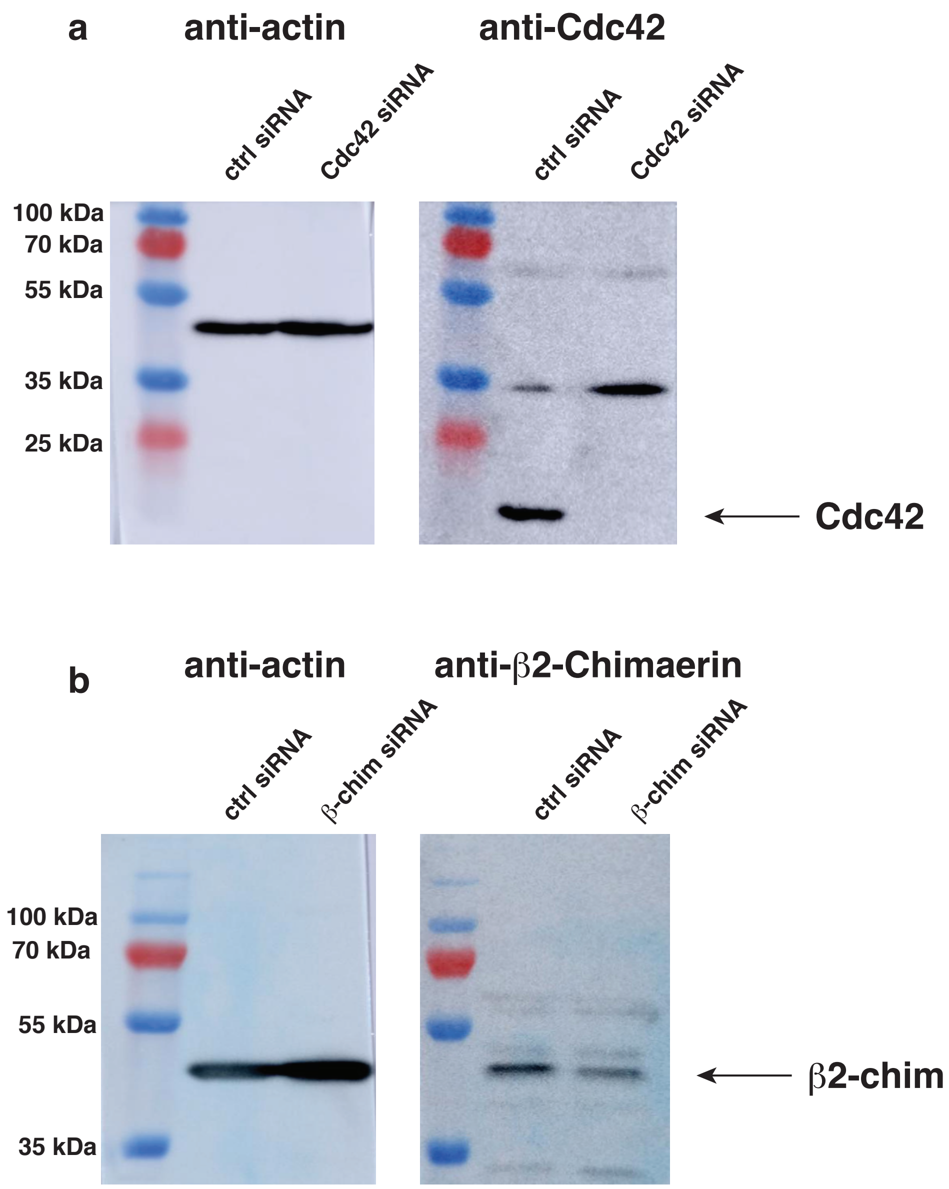

Supplementary Figure 6: siRNA-mediated depletion of Cdc42 and β2-chimaerin verified by Western blot.

Total cell extracts from HeLa cells treated with Cdc42-directed (a) or β2-chimaerin-directed siRNA (b) were tested by Western blot and compared to cell extracts from cells treated with control siRNA. Actin : 42 kDa, Cdc42: 22kDa, β2-Chimaerin : 54kDa.

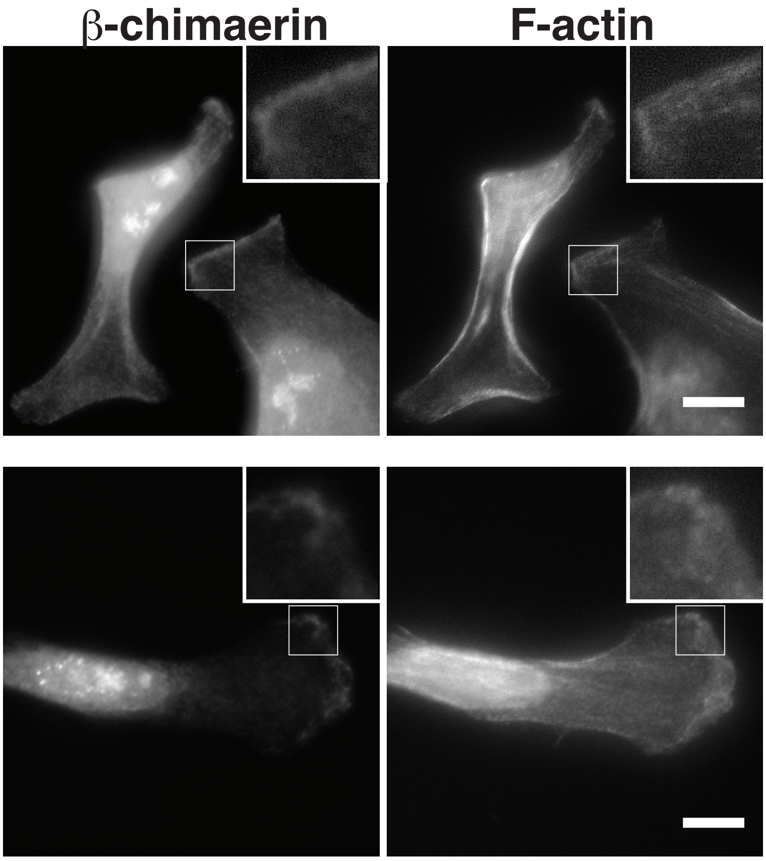

Supplementary Figure 7: Colocalization of β2-chimaerin with F-actin at the edge of lamellipodia

Hela cells were stained with anti-β2-chimaerin antibody and phalloidin. Insets show zoomed regions of the cell edge. Scale bars : 10 µm.

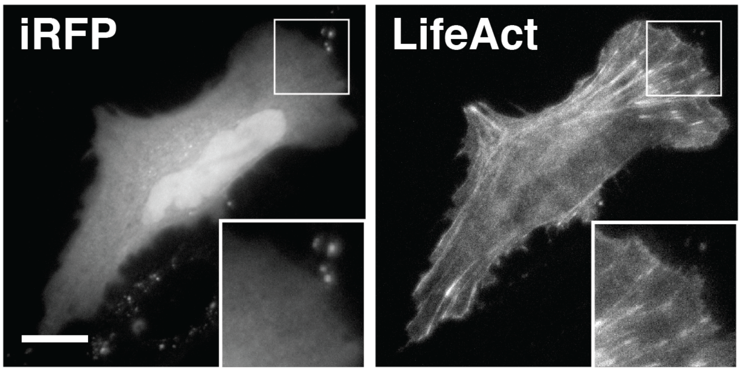

Supplementary Figure 8: control of volume effects affecting fluorescence in protruding regions of the cell
HeLa cells expressing free cytosolic iRFP together with LifeAct-GFP. Insets show zoomed regions of the cell edge. No enrichment was observed at the tip of protruding edges. Scale bars: 10 µm.

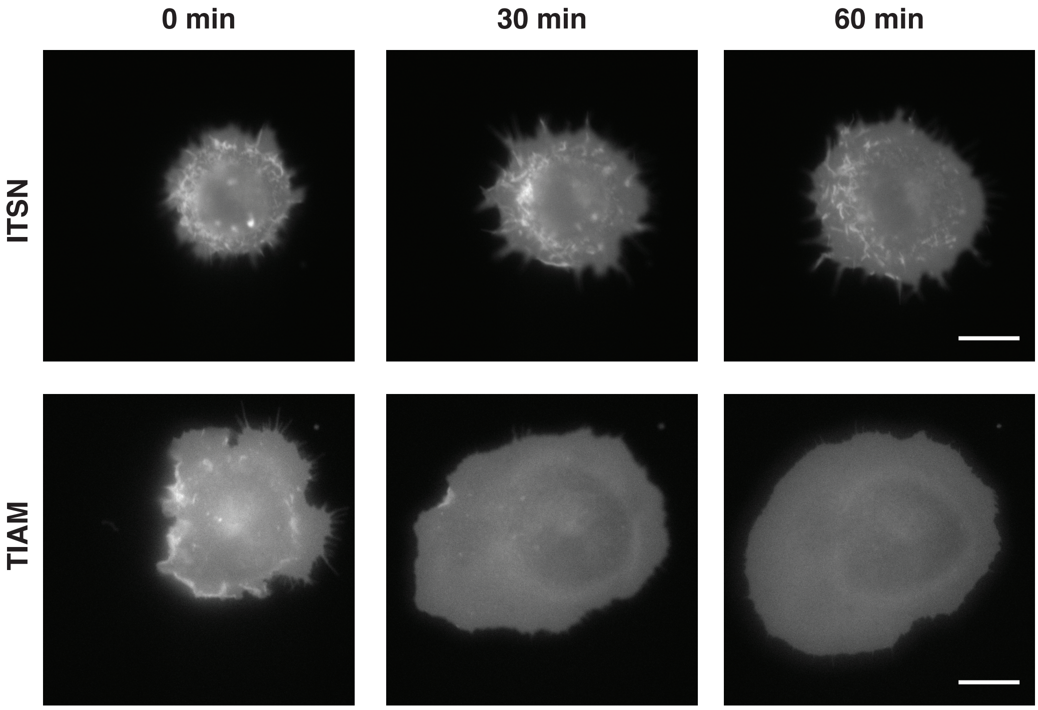

Supplementary Figure 9: ITSN and TIAM activation trigger different cytoskeletal responses
Hela cells expressing CIBN-GFP-CAAX together with ITSN-CRY2-mCherry (top) or TIAM- CRY2-mCherry (bottom) were illuminated with 2x gradients for indicated times. Cells were released from micropatterns with BCN-RGD at time 0 of stimulation. Cell membrane was visualized by TIRF microscopy using the GFP channel. While ITSN activation induced the formation of numerous filopodia, TIAM activation induced mostly lamellipodial protrusions. Scale bars: 10 µm.

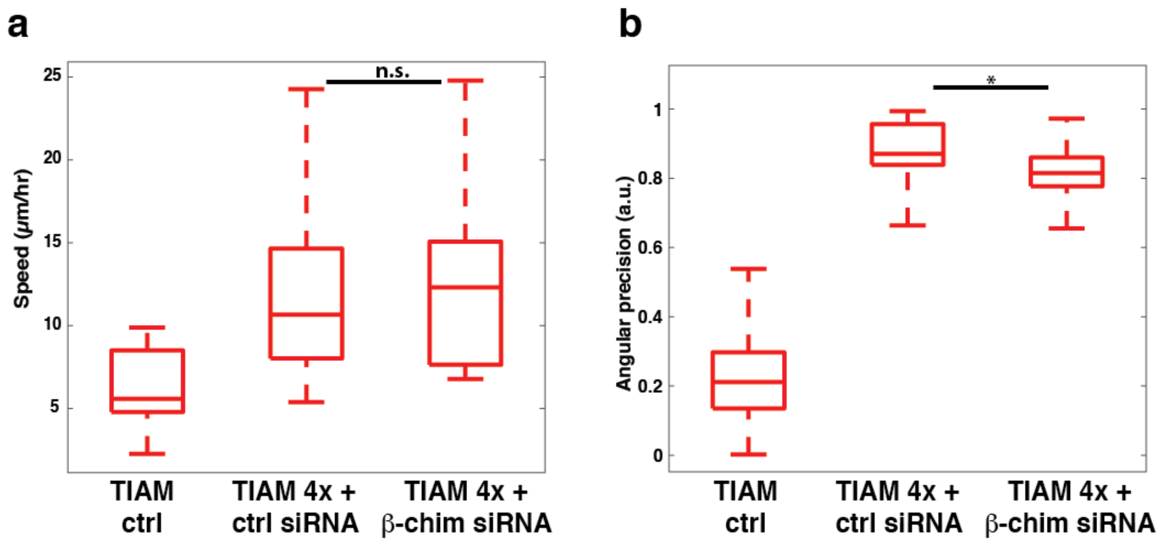

Supplementary Figure 10: effect of β2-chimaerin depletion on TIAM-induced cell migration
Cells expressing CIBN-GFP-CAAX together with TIAM-CRY2-mCherry were stimulated with control (no gradient, n=19) or 4x gradient as in Figure 6 after treatment with control (n=15) of β2-chimaerin-directed siRNA (n=13), and cell movement was monitored. ***(a)*** *Box plots showing the instantaneous speed of cells.* ***(b)*** *Angular precision of cell displacement. n.s.: non significant (p>0.05).*:p<0.05* (Wilcoxon rank sum test).
