# Supplementary material for: Optogenetic dissection of Rac1 and Cdc42 gradient shaping
Source: Nat Commun. 2018 Nov 16;9:4816. doi: 10.1038/s41467-018-07286-8 (PMC6240110; doi:10.1038/s41467-018-07286-8)
Supplement: Supplementary file 3 — Description of Additional Supplementary Files [file 41467_2018_7286_MOESM3_ESM.docx]

**Description of Additional Supplementary Files**

File Name: **Supplementary Movie 1: PAK1 recruitment following ITSN activation with 4x gradients**

Description: Hela cells expressing CIBN-GFP-CAAX, ITSN-CRY2-mCherry and PAK1-iRFP were adhered on round microppatterns. Membrane recruitment of PAK1-iRFP following 4x activation gradients was recorded using TIRFM, initial fluorescence was subtracted for normalization, and fluorescence was averaged over n=15 cells.

File Name: **Supplementary Movie 2: PAK1 recruitment following TIAM activation with 4x gradients**

Description: Hela cells expressing CIBN-GFP-CAAX, TIAM-CRY2-mCherry and PAK1-iRFP were adhered on round microppatterns. Membrane recruitment of PAK1-iRFP following 4x activation gradients was recorded using TIRFM, initial fluorescence was subtracted for normalization, and fluorescence was averaged over n=15 cells.

File Name: **Supplementary Movie 3: examples of cell movement induced by ITSN activation with various illumination gradients**

Description: Hela cells expressing CIBN-GFP-CAAX and ITSN-CRY2-mCherry were adhered on APP round micropatterns. Cells were released from micropatterns with BCN-RGD and immediately stimulated with various illumination gradients (depicted on left column). Duration after release from the patterns and beginning of the stimulation is indicated at the bottom right of the movie. Fluorescence from CIBN-GFP-CAAX was recorded using TIRFM. 6 cells are shown for each condition. Initial positions are shown with vertical lines.

File Name: **Supplementary Movie 4: examples of cell movement induced by TIAM activation with various illumination gradients**

Description: Hela cells expressing CIBN-GFP-CAAX and TIAM-CRY2-mCherry were adhered on APP round micropatterns. Cells were released from micropatterns with BCN-RGD and immediately stimulated with various illumination gradients (depicted on left column). Duration after release from the patterns and beginning of the stimulation is indicated at the bottom right of the movie. Fluorescence from CIBN-GFP-CAAX was recorded using TIRFM. 6 cells are shown for each condition. Initial positions are shown with vertical lines.
